# Choosing Appropriate Theories for Understanding Hospital Reporting of Adverse Drug Events, a Theoretical Domains Framework Approach

**Published:** 2018

**Authors:** Gloria Shalviri, Bahareh Yazdizadeh, Fariba Mirbaha, Kheirollah Gholami, Reza Majdzadeh

**Affiliations:** a *Knowledge Utilization Research Center, Tehran University of Medical Sciences, Tehran, Iran. *; b *Research Center for Rational Use of Drugs, Faculty of Pharmacy, Tehran University of Medical Sciences, Tehran, Iran.*; c *Knowledge Utilization Research Center; Epidemiology and Biostatistics Department, School of Public Health, Tehran University of Medical Sciences, Tehran, Iran.*

**Keywords:** Adverse drug event, Pharmacovigilance, Theoretical domains framework, Theory selection, Medication error, Reporting

## Abstract

Adverse drug events (ADEs) may cause serious injuries including death. Spontaneous reporting of ADEs plays a great role in detection and prevention of them; however, underreporting always exists. Although several interventions have been utilized to solve this problem, they are mainly based on experience and the rationale for choosing them has no theoretical base. The vast variety of behavioural theories makes it difficult to choose appropriate theory. Theoretical domains framework (TDF) is suggested as a solution. The objective of this study was to select the best theory for evaluating ADE reporting in hospitals based on TDF. We carried out three focus group discussions with hospital pharmacists and nurses, based on TDF questions. The analysis was performed through five steps including coding discussions transcript, extracting beliefs, selecting relevant domains, matching related constructs to the extracted beliefs, and determining the appropriate theories in each domain. The theory with the highest number of matched domains and constructs was selected as the theory of choice. A total of six domains were identified relevant to ADE reporting, including “Knowledge”, “Skills”, “Beliefs about consequences”, “Motivation and goals”, “Environmental context and resources” and “Social influences”. We found theory of planned behavior as the comprehensive theory to study factors influencing ADE reporting in hospitals, since it was relevant theory in five out of six relevant domains and the common theory in 55 out of 75 identified beliefs. In conclusion, we suggest theory of planned behavior for further studies on designing appropriate interventions to increase ADE reporting in hospitals.

## Introduction

Early detection and reporting adverse drug events (ADEs) is a necessary component for improving patient safety. Drug-related complications may cause serious injuries including death. ADEs have been recognized as the fourth to sixth leading cause of death in the United States (1). World Health Organization (WHO) defines ADEsas“any untoward medical occurrence that may present during treatment with a pharmaceutical product but which do not necessarily have a causal relationship with this treatment” (2). ADEs involve adverse drug reactions (ADRs) and medication errors (MEs). According to WHO, an ADR is “a response which is noxious and unintended, and which occurs at doses normally used in humans for the prophylaxis, diagnosis, or therapy of disease, or for the modification of physiological function” (2). ME is “any preventable event that may cause or lead to inappropriate medication use or patient harm while the medication is in the control of the health care professional, patient, or consumer. Such events may be related to professional practice, health care products, procedures, and systems, including prescribing, order communication, product labeling, packaging, and nomenclature, compounding, dispensing, distribution, administration, education, monitoring, and use.” (3). 

The science and activities related to the detection, assessment, understanding and prevention of adverse effects or any other drug-related problem is called pharmacovigilance (2). Evidence from pharmacovigilance has revealed that spontaneous reporting of ADEs by health care professionals plays a great role in detection and prevention of ADEs (4). Spontaneous reportingre requires health care professionals to complete designated ADE reporting forms when they suspect that an ADE has occurred, and submit them to a national pharmacovigilance center. Spontaneous reportingis a basic method for detecting ADEs after marketing medicines. Along with several advantages, spontaneous reporting has some disadvantages too. One of them is underreporting (5). Studies reported from developed countries revealed that only 2-4% of all ADEs and 10% of serious ones are reported (6). Several interventions have been utilized implemented to improve spontaneous reporting of ADEs. Some of these interventions include educational interventions (7), electronic ADR reporting system (8), placing ADR reporting forms in patient charts (9), considering incentive for ADE reporters (10), and giving feedback to reporters (11). However, these interventions are mainly selected based on experience and the rationale for choosing them has no theoretical base. Furthermore, interventions applied in one setting may not be appropriate for all health settings and there is a need for stronger evidence that guides selection of relevant and comprehensive interventions.

There are often perceived barriers which make it difficult for health care practitioners to modify their past behavior after initiation of a behavioral intervention. Designing interventions aimed at behavior change requires comprehensive understanding of the target behavior among health workers. Theory-based evaluation of a target behavior guides identification of obstacles to behavior change. If behavior changing interventions are designed based on these identified barriers, then they may be more successful (12, 13). 

Spontaneous reporting of ADEs has been implemented in Iran by Iranian pharmacovigilance center (IPC) since 1998. IPC has received more than 40000 reports of ADEs from health care professionals around the country since 1998. However, like as any other national pharmacovigilance center, the center faces the problem of underreporting. As a primary step to promote hospital reporting of ADEs, IPC issued a guideline for reporting ADEs to each hospital had to designate a health care professional (mainly nurses) as a responsible person for collecting and reporting ADEs to IPC. According to the guideline, however, health care professionals are free to submit observed ADEs directly to IPC if they prefer not to report via the ADE person in the hospital. Despite these interventions, the problem of ADE underreporting still exists and many ADEs are notreported. ADE reporting behavior is not fully institutionalized in many hospitals.

Several theories have been utilized for understanding behavior in health settings. These theories have been used to explain a behavior, predicting which behavior constructs are susceptible to change, and identifying barriers to behavior change and also finding ways to change the behavior. Some theories focus on factors related to individual professionals (e.g. cognitive theories or theory of planned behavior); some concentrate on social context and interactions; the others target economical and organizational context. This variety in theories makes it difficult to choose appropriate theory for a study. Theoretical domains framework (TDF) is suggested by a group of experts as a solution to the problem of theory selection (14). This framework consists of 12 domains which are extracted from 128 explanatory constructs belonging to 33 psychological theories on behavior change. These domains include knowledge, skills, social/professional role and identity, beliefs about capabilities, beliefs about consequences, motivation and goals, memory, attention and decision processes, environmental context and resources, social influences, emotion, behavioral regulation, and also nature of the behaviors. Questions related to each of the twelve domains are suggested. The effective constructs on behavior change are determined by conducting a qualitative analysis (e.g. focus group discussion, interview) and related domains are selected to choose appropriate theory for changing behavior.

In this study we considered hospital reporting of ADEs as the target behavior. The aim of this study was to select the best theory of behavior change to improve ADE reporting, based on TDF approach. 

## Experimental

*Design*

This was a qualitative study carried out by conducting three different focus group discussions (FGDs). The FGD participants included pharmacists and nurses working at Tehran hospitals. The study was approved by the Institutional Review Board of Tehran University of Medical Sciences which follows the Helsinki Declaration. 

*Participants*

The method of sampling in this study was purposeful sampling by maximum variation. We identified and invited 30 nurses and 15 pharmacists from the IPC list of hospitals in Tehran. There were 117 Drug Safety Officers (DSOs) in the IPC list of DSOs in hospitals of Tehran from 117 hospitals, among them 60 were nurses, 51 pharmacists and six physicians. In order to get broad information about barriers to ADE reporting, participant nurses were selected from two different groups. One group involved those who were introduced to IPC as DSOs in each hospital and the other group consisted of nurses who didn’t have this responsibility. The pharmacists group involved only DSOs in the hospitals, because pharmacists not responsible for ADE reporting had not reported considerable number of ADEs to IPC. In order to capture broader range of ideas among the study participants, study sample included different geographical regions in Tehran, different hospital sizes, different hospital settings (teaching versus non-teaching hospitals), and hospitals with different reporting number of ADEs.

*Materials*

We selected a set of questions related to 12 TDF domains which was suggested by the framework. These questions were first previewed by conducting interviews with one nurse and one pharmacist in two different hospitals. According to their answers and comments, we modified the questions to make them clearer. A final version of questions, including the main questions and prompts, was prepared to guide the FGDs. At the end of each section in the FGDs, the participants had the opportunity to add any additional comments not mentioned during the discussions. 

*Procedure*

A formal invitation letter, including the purpose of the study and the ethical considerations, was sent to the identified nurses and pharmacists based on the criteria mentioned above. Verbal informed consent was obtained and recorded from all participants at the beginning of each FGD. All three FGDs were facilitated by the same group of researchers, including an epidemiologist expert in knowledge translation and exchange (KTE), a medical sociologist, and a pharmacist expert in pharmacovigilance. One of these persons took notes during discussions. The anonymity of the participants was maintained in the transcripts. Each FGD lasted about 90 min. 

*Analysis*

The analysis was performed through five steps including coding FGD transcript, extracting beliefs, selecting relevant theoretical domains, matching related constructs to the extracted beliefs, and determining the appropriate theories in each domain. Each step is described below in detail.

*Coding FGD transcript*

Two of the researchers reviewed the transcripts of all performed FGDs independently and deductively coded them into the 12 theoretical domains. Also, the transcripts were inductively analyzed by coders in order to identify any additional themes not included in TDF. A number of themes and sub-themes were detected. The two researchers had high agreement on extracted themes and subthemes, and the disagreements were resolved through discussions.

*Extracting beliefs*

Two researcher extracted specific beliefs from the detected sub-themes in step one. A belief was defined as “a set of responses relating to a unique theme which described a special problem and/or the impact of that belief on the target behavior” (15).

*Selecting relevant theoretical domains*

Three researchers reviewed the extracted beliefs within each theoretical domain. The theoretical domains with potential barriers for behavior change were considered as relevant. The relevant theoretical domains needed to have the following criteria, as well (15):

(1) Contradictory beliefs were expressed.

(2) Impact of a specific belief on the target behavior was shown by evidence. 

Those theoretical domains which were not recognized as relevant domains were excluded from the study.

*Matching related constructs to the extracted beliefs*

The aim of this step was to match relevant constructs to each identified belief. So the same three researchers were provided with a list of TDF constructs (14) belonging to each related domain (16). They independently matched related constructs from the provided list to the specific beliefs in each domain. The final selection of constructs for each specific belief was determined based on agreement between the coders. The construct with higher frequency of matching by coders was selected for each specific belief. 

*Determining the appropriate theories in each domain*

The research team selected appropriate theories in each relevant domain, among those represented in TDF approach, through discussion and consideration of previous similar methodological studies (15, 17). The researchers could choose more than one theory for each domain if they observed constructs from those theories.

*Theory selection based on coding the constructs of the relevant domains*

We calculated the total number of domains and construct that matched to an individual theory. The theory with the highest number of matched domains and constructs was selected as the theory of choice for the purpose of this study.

## Results

According to different steps of the analysis, the result of our study is as following:

*Step one coding FGD transcript*

FGD analysis and detected barriers, has previously been published in detail in another paper (18).Here we present the result of theory selection based on TDF approach.

*Step two and three: extracting beliefs and selecting relevant theoretical domains*

A total of six domains were identified as relevant domains to ADE reporting. These domains included “Knowledge”, “Skills”,“Beliefs about consequences (Anticipated outcomes/attitude)”, “Motivation and goals (Intention)”, “Environmental context and resources”, and “Social influences (Norms)”.We identified 75 specific beliefs relevant to these six domains.“Beliefs about consequences (Anticipated outcomes/attitude)” and “Skills” were associated with the highest and lowest number of beliefs, respectively.

*Step four: matching related constructs to the extracted beliefs*

Those constructs that were detected as relevant to the extracted beliefs in each domain are mentioned in table 1. All or at least two out of the three coders were in agreement upon matching appropriate constructs to the identified six relevant domains. 

*Step five determining the appropriate theories in each domain*

The suggested relevant theories in this study included Knowledge, attitude, and behavior (KAB) model for examining “knowledge” domain; Theory of planned behavior (TPB) and Operant learning theory (OLT) for studying “Beliefs about consequences” domain; TPB, Social cognitive theory (SCT) and Personal project approach (PPA) for evaluating “Motivation and goal” domain; TPB and Normative model of work team effectiveness (NMWTE) for assessing “Social influences” domain; and TPB for studying “Skills” and “Environmental context and resources” domains.

*Step six: theory selection based on coding the constructs of the relevant domains.*

The numbers of beliefs in each domain and relevant theories to evaluate the identified constructs are shown in table 2. We picked TPB as the comprehensive theory to study factors associated with ADE reporting behavior in hospitals because of the following reasons:

1. TPB was identified as a relevant theory in five out of six relevant domains in this study. 

2. TPB was the only theory of choice in two domains (“Skills” and “Environmental context and resources”). 

3. TPB was the common theory in 55 out of 75 identified beliefs.

*Reasons for choosing the domains relevant to ADE reporting in hospitals*

The “knowledge” domain was identified as relevant since there were contradictory beliefs in this area. Identified beliefs on knowledge about ADE reporting procedure and guideline were in conflict. Some declared that they were aware of existing guidelines and procedure for reporting ADEs, but others referred to lack of knowledge in this area as one of the barriers to ADE reporting. Furthermore, we found that misunderstanding definitions of ADR and ME, along with lack of knowledge about what should be reported, could have an impact on target behavior. As an example, some participants did not believe they should report non-serious ADEs, uncertain ADEs and/or non-preventable ones. However, these beliefs were in contrast with those of others that considered any type of observed ADEseligible for reporting.

We decided to select “Skills” domain as a relevant domain because some participants expressed ADE reporting as an easy procedure and the others mentioned it difficult. Most participants emphasized that reporting MEs, as a part of ADEs, were much more difficult than reporting ADRs. This contradiction in perceived difficulty of ADE reporting made us consider this domain for further investigation.

The domain of “Beliefs about consequences (Anticipated outcomes/attitude)” consisted of 21 specific beliefs. Although there was a general agreement on positive outcomes of ADE reporting, some individuals believed that ADE reporting could also have some negative consequences such as criticism and/or punishment of the reporter or those involved in the reported MEs. This led us to select this domain for further review.

**Table 1:** Relevant constructs to the extracted beliefs in each domain.

**Domain**	**Relevant constructs detected by coders**
Knowledge	“Knowledge”, “Schemas, mindsets and illness representations”, “Procedural knowledge” and “Knowledge about condition/scientific rationale”
Skills	“Skills” and “Competence/ability/skill assessment”
Beliefs about consequences (Anticipated outcomes/attitude)	“Consequences”,“Outcome expectancies” and “Reinforcement/punishment/consequences”
Motivation and goals (Intention)	“Intention; stability of intention/certainty of intention”, “Goal priority” and “Commitment”
Environmental context and resources	“Resources/material resources (availability and management)” and “Environmental stressors”
Social influences (Norms)	“Social/group norms: subjective, descriptive, injunctive norms”, “Social pressure”, “Team working”, “Management commitment”, “Social support”, “Social/group norms”,

**Table 2:** The number of beliefs in each domain and identified relevant theories

**Domain**	**Number of identified beliefs**	**Identified theories**
Knowledge	20	KAB^1^
Skills	5	TPB^2^
Beliefs about consequences	21	TPB and OLT^3^
Motivation and goals	8	TPB, SCT^4^ and PPA^5^
Environmental context and resources	12	TPB
Social identities (Norms)	9	TPB and NMWTE^6^

(1) Knowledge, attitude, behavior; (2) Theory of planned behavior; (3) Operant learning theory; (4) Social cognitive theory; (5) Personal project approach; (6) Normative model of work team effectiveness.

We considered “Motivation and goals (Intention)” as one of the relevant domains to hospital ADE reporting because of contradictory beliefs mentioned by participants. Most FGD participants believed that they were determined to report ADEs in hospitals. However, some individuals outlined a few reasons which made them decide not to report observed ADEs. Lack of feedback from IPC and lack of incentives were suggested by participants as reasons for their low motivation.

“Environmental context and resources (Environmental constraints)” was another domain considered as relevant, based on the study criteria. This domain contained12 identified specific beliefs which described physical and resource factors facilitating or hindering ADE reporting at hospitals. Many FGD participants explained that physical and resource factors can influence target behavior. They listed several barriers to reporting ADEs in hospitals such as heavy workload and lack of necessary equipment (such as fax machines) for reporting ADEs. Also, they reported that time pressure affected the intent to report ADEs. We concluded that there were lots of factors in this domain which might impact hospital reporting of ADEs. So, we added this domain to the list of selected relevant domains.

The last domain judged to be influential on target behavior was “Social influences (Norms)”.We found some conflicts in participants’ answers to the questions about involvement of hospital managers and other health care professionals in ADE reporting, and getting support from them to report ADEs. According to the participants, hospital managers approved ADE reporting; however, they did not do anything to facilitate it. Some FGD attendees believed that there were no individuals or groups in their place of work that disapproved ADE reporting while others noted that some individuals might oppose reporting because of time constraints or fear of criticism and punishment. However, in their opinion, opponents to ADE reporting might not readily disclose their disapproval. Moreover, they pointed out that lack of collaboration between nurses, physicians, and pharmacists, i.e. poor teamwork, could be considered as a significant barrier to reporting ADEs. These findings guided us to select this domain as another influential one for further studies.

*Reasons to exclude non-relevant domains to ADE reporting in hospitals*

Among twelve domains suggested by TDF approach, we identified 6 domains as non-relevant to hospital reporting of ADEs. These domains included “Social/professional role and identity”; “Beliefs about capabilities”; “Memory, attention and decision processes”; “Emotion”; “Behavioral regulation”; and “Nature of the behaviors”.

All FGD participants confirmed ADE reporting as their professional responsibility and moral obligations. None of the participants considered reporting of an ADE by a colleague as an act against them. So, we did not select “Social/professional role and identity” as a relevant domain to ADE reporting in hospitals.

We did not consider “Beliefs about capabilities” as a relevant domain because all participants stated that they were confident in their abilities to report ADEs despite many difficulties. Also all claimed that difficulties of ADE reporting were related to other domains and none of them could be attributed to participants’ self-efficacy in reporting ADEs.

“Memory, attention, and decision processes” domain was also excluded from the list of influential domains on ADE reporting since all participants mentioned that decision making for ADE reporting was easy to them. They emphasized that they never forgot to report the observed ADEs. Also, they stressed that they never initially decided not to report observed ADEs since they considered reporting as a professional responsibility.

Participants mentioned that their ADE reporting was never influenced by emotional factors. To them, emotional factors were neither facilitators nor obstacles to ADE reporting. Therefore, we did not add “Emotion” to the list of relevant domains to change the target behavior in this study.

FGD participants had several recommendations for encouraging ADE reporting; however none of them referred to the constructs of “Behavioral regulation” domain. So, we did not choose this domain as a relevant domain. Similarly, “Nature of the behavior” domain was not selected as relevant because the properties of ADE reporting as the target behavior were well explained by the respondents. 

## Discussion

Applying TDF approach to the beliefs derived from statements of FGD participants enabled us to identify six relevant domains to ADE reporting in hospitals including “Knowledge”, “Skills”, “Beliefs about consequences (Anticipated outcomes/attitude)”, “Motivation and goals (Intention)”, “Environmental context and resources” and “Social influences (Norms)”. Also, the findings of this study revealed that a total of six theories could be considered as appropriate theories for evaluating barriers to reporting ADEs in hospitals (table 2).Except for “knowledge” domain, which is not a TPB construct, the other five identified relevant domains in this study could be evaluated by applying TPB. The large number of identified beliefs in “knowledge” domain; however, suggested that this domain may have a significant impact on the target behavior and it is necessary to investigate it besides constructs belonging to the TPB. To the best of our knowledge, this is the first study that applies TDF approach to select appropriate theory for evaluating ADE reporting behavior in hospitals.

*Suggested criteria for selecting the right theory*

There are numerous theories for explaining, predicting, or changing behavior; each of them focuses on specific factors influencing behavior, such as individual or organizational factors. Because of this vast variety, it is important to know and follow the best criteria for choosing the most relevant theory to examine a behavior. The selection process of appropriate theory to study behaviors of health care providers has been described in some studies. Graham *et al*. provided a list of planned action theories and analyzed them based on their similarities and differences (19). They explained that different theories addressed different action plans; so it appeared necessary to determine how a theory coded into action categories in the theory selection process. The authors recommended that careful evaluation of the theory components, as well as ensuring suitability of the theory for the target context and culture, were the necessary steps towards theory selection.

Glanz argued that criteria for selection of appropriate theory should not be based on researchers’ interest or some specifications of the theory such as being conventional, novel, or user friendly. They suggested three criteria as necessary considerations while choosing a suitable theory including “its logic or internal consistency in not yielding the mutually contradictory derivations; the extent to which it is parsimonious, or broadly relevant, while using the manageable number of concepts; and its plausibility in fitting with prevailing theories in the field” (20).Michie *et al.*designed a consensus approach as a response to the lack of a systematic approach for choosing appropriate theories (14). They introduced TDFas their systematic method for theory selection. 

There are two published studies which applied TDF approach for choosing the appropriate theory. Francis *et al*. applied TDF for evidence-based selection of theories to understand clinicians’ blood transfusion behavior in the United Kingdom (17). They identified 5 relevant theoretical domains to the studied target behavior including “knowledge”, “beliefs about capabilities”, “beliefs about consequences”, “social influence” and “behavioral regulation”. They reported seven theories as appropriate for that context and concluded that TDF approach is beneficial in selecting theories and could result in broader potential explanations in comparison with a single theoretical model. 

Islam *et al*. also conducted a study to choose relevant theories for predicting transfusion behavior of Canadian intensive care physicians (15). Although the target behavior was the same in these two studies, the findings of the second study revealed seven relevant domains, four of which were the same as the previous study. Using the same methodology, the authors identified similar theories in all recognized relevant domains as the UK study, but UK study authors added Personal Project Approach (PPA) as an identified theory under “motivation and goals” domain. The authors concluded that applying TDF to theory selection was a useful method as a systematic approach and could provide a rationale for designing appropriate interventions. The differences in identified beliefs, relevant domains, and theories in these two studies indicated that it was necessary to consider the context for identification of behavioral change barriers and needed interventions. Health care professionals could express different views and beliefs toward the same behavior in different contexts. TDF was a useful approach to explore those differences and resulted in identifying appropriate theories in each setting. 

*Strengths of selecting the appropriate theory based on the TDF approach*

Based on the results of this study, we can categorize strengths of applying TDF approach to the process of selecting appropriate theory for changing behavior into the following points:

First, we discovered that TDF approach was highly compatible with necessary components of Glanz’s criteria for choosing right theory. In the current study, TDF approach helped us provide a rationale for choosing six theories from33 psychological theories on behavior change. So Glanz’s first assumption, its logic or internal consistency in not yielding the mutually contradictory derivations, was met. We believe that the wide spectrum of psychological theories (33 theories) and related constructs (128 explanatory constructs) included in TDF approach, diminish the possibility of overlooking eligible theories in the theory selection process and could help provide a comprehensive list of theories to consider. 

In addition, TDF approach enabled us to recognize the common theory (TPB) among five identified relevant domains as the potential theory of choice so the condition for Glanz’s second assumption, the extent to which it is parsimonious, or broadly relevant, while using the manageable number of concepts, could be provided. 

Godin *et al*. conducted a systematic review on 78 studies based on social cognitive theories (21). The authors of this systematic review concluded that TPB was the most relevant theory to study healthcare professionals’ behaviors. So applying TDF approach in this study, led us to have TPB as one of the most relevant and prevailing theories in hand as the theory of choice. This is in accordance with the third criterion for theory selection mentioned by Glanz as “its plausibility in fitting with prevailing theories in the field”. 

Our findings demonstrate one of the other advantages of TDF approach that is the possibility of providing a semiquantitative tool for comparing identified relevant domains, by calculating the number of detected relevant beliefs in each determined domain (table 2). This will increase the researchers’ ability to choose the theory which includes greater number of health care providers’ beliefs. However, the process of theory selection should not be entirely based on quantitative comparison of the identified beliefs and the importance of determined beliefs should also be considered. 

The findings of this study showed that applying TDF approach might provide more than one theory to investigate each relevant domain. For instance, TPB and OLT were mapped to domain “beliefs about consequences”; TPB, SCT, and PPA matched to domain “motivation and goals”; and TPB and NMWTE mapped to domain “social identities”. This characteristic is another advantage of the TDF approach because it provides more choices of theories based on the target culture and context.

Finally, the systematic approach of TDF presents a step-by-step theory selection process which provides opportunities to repeat the study if desired, although the repeatability needs more objective process. The findings of our study are consistent with both UK and Canadian studies (15, 17) with respect to general advantages of TDF approach.

*Limitations of selecting the right theory based on TDF approach*

We faced few limitations while conducting our study. The first limitation was that the rationale for considering some constructs in TDF approach was not clear. This problem appears to be highly related to lack of access to a clear definition of the 12 domains involved in TDF approach. As an example, the construct “perceived behavior control” is related to the domain “beliefs about capabilities” and is defined as “an individualʹs perception of the ease or difficulty of performing the behavior of interest” (16). The perceived difficulty of performing the target behavior may be attributed to control beliefs over both situational and internal factors to inhibit or facilitate performing the behavior (22). This construct has two different components including self efficacy and controllability. However the general definition of the domain “Beliefs about capabilities” is not clearly mentioned in TDF approach to explore if the domain target control beliefs originated from internal factors such as self efficacy, external factors such as facilitators or obstacles, or both of them. This situation was worse when an individual construct belonging to more than one domain, e.g. ‘self efficacy” is listed under more than one domain.

Another limitation was the subjective process of matching appropriate construct to each specific belief. We tried mitigating this condition by providing coders with a list of construct definitions. Similarly the process of theory selection for each domain had limited objectivity. 

Lastly, a well defined list of constructs in each psychological theory involved in TDF approach was missing, making it impossible to provide a matrix of theories and their constructs as an objective tool for facilitating the theory selection process. We tried to manage this limitation through discussion among researchers and also by following similar previous studies (15, 17).

The similar UK and Canadian studies revealed some limitations as well, such as lack of inter-rater reliability in the UK study, and lack of clarity in the definitions of the theoretical domains. 

## Conclusion

The knowledge about safety of marketed medicines is continuously changing and new evidence is always emerging while a medicinal product is on the market. So every medicinal product is still under trial, even if it has been approved for marketing. Spontaneous reporting of ADEs by health care professionals to a national pharmacovigilance center is a well known methodology for increasing knowledge about drug safety. However, the problem of underreporting exists. The continuous generation of new evidence about drug safety puts ADE reporting behavior of health care professionals in a complex condition. So, it is necessary to understand ADE reporting behavior of health care professionals using a well- designed theoretical approach. We applied TDF approach as a systematic method to identify most relevant theoretical domains to ADE reporting behavior and consequently most appropriate theory for evaluating this behavior. Based on our study, TPB was the theory of choice for examining the ADE reporting behavior in hospitals. The findings of our study indicated that TDF is an appropriate approach for determining appropriate domains and theories for evaluating the target behavior. The results of this study confirmed that TDF could be considered as a comprehensive systematic approach for choosing appropriate theories. However, further studies are needed to alleviate limitations of using this method as a theory selection approach. Based on the result of this study, we have conducted another study to apply TPB to investigate perceived barriers against ADE reporting in hospitals.
